# Mitigating the Effect of Climate Change within the Cereal Sector: Improving Rheological and Baking Properties of Strong Gluten Wheat Doughs by Blending with Specialty Grains

**DOI:** 10.3390/plants12030492

**Published:** 2023-01-21

**Authors:** Rubina Rumler, Denisse Bender, Regine Schoenlechner

**Affiliations:** Department of Food Science and Technology, Institute of Food Technology, BOKU—University of Natural Resources and Life Sciences Vienna, Muthgasse 18, 1190 Vienna, Austria

**Keywords:** wheat blends, gluten-free cereals, baking, rheology, climate change

## Abstract

Due to the effect of climate change, wheat flour qualities with extremely high dough extensibility or dough strength are becoming more common, which impairs the production of selected wheat products such as pastries. The aim of this study was to investigate the effect of sorghum, millet, amaranth, or buckwheat addition to such a strong gluten common wheat flour (*Triticum aestivum*) on its rheological and baking properties. Raw materials were analyzed chemically (ash, protein, fat, starch, total dietary fiber) and physically (water absorption index, water solubility index, and pasting properties). Selected rheological analyses (Farinograph® and Extensograph^®^) were carried out on wheat blends, including up to 30% alternative grains. The baking properties of the blends were evaluated on standard bread and sweet milk bread recipes. Results showed that low amounts (5%) of sorghum and millet improved the dough stability of the high-gluten wheat flour. For optimum dough extensibility, additions of 30% sorghum, 15% millet, or 20% amaranth were needed. The use of gluten-free grains increased bread volume and decreased crumb firmness of the sweet milk breads when added at lower levels (5–15%, depending on the grain). In conclusion, cereal blending is a supportive tool to mitigate the effects of ongoing climate change and can enhance biodiversity and nutrition.

## 1. Introduction

In recent years the interest in bakery products made from cereal blends has increased for various reasons. Cereal blending can offer health-promoting benefits as it can diversify, complement, or even enhance the nutritional properties (e.g., secondary plant metabolites and dietary fibers) of flour. This allows the development of end-products with higher nutritional quality, as was shown for, e.g., in maize tortillas fortified with amaranth, which then possessed significantly higher antioxidative activities [1], or for the addition of finger millet to wheat biscuits, which improved the fiber, calcium, iron, and zinc content [2]. In general, data from several sources have identified the health potential of alternative cereals, such as buckwheat [3] or sorghum [4], in food products. Secondly, the blending of cereals in bakery products can also have economic reasons. For instance, wheat is not readily available in large quantities in Africa, yet the demand for wheat products is high. Therefore, adding local cereals (e.g., sorghum) to wheat can lower the import requirements of wheat.

In Europe, wheat is the most important cereal in the food industry. Its uses are most evident within the baking industry, in a broad variety of products, ranging from bread, cakes, and pastries to waffles or cookies.

Wheat is characterized by its excellent baking properties, resulting from the unique properties of the gluten protein fraction, which gives the dough a firm but extensible and elastic character. However, the ongoing climate change has become a challenging factor for the wheat cereal industry in recent years. On the one hand, intensely hot and dry summers have led to a decline in wheat yields. On the other hand, the wheat protein quality and amounts (in particular gluten) have been observed to vary greatly depending on the climatic conditions, as reported by Gagliardi et al. [5].

At this point, it has to be stressed that high protein quantities have principally been valued by the baking industry and, thus, the wheat breeding industry. Harvested Austrian wheat can generally be considered to be of superior quality; they are also sought after and exported to many countries. Yet for some products, such superior wheat qualities are less desirable, as excessive dough extensibilities or dough strengths are not suitable for all products. Some require lower wheat qualities, in particular fine bakery products such as cakes and waffles and other fine bakery products that are made from batter rather than from dough. So far, only a minimal amount of data is available that describes in detail such climatic effects on wheat quality. Wrigley et al. [6] pointed out that the wheat quality was strongly dependent on the weather conditions, particularly dough strength seemed to be affected by increased temperatures. Since the effects of climate change will continue to be an ongoing issue, the cereal industry is now compelled to respond in order to keep the quality of the existing wheat products constant [7].

Possible solutions may be the use of food additives to adjust the wheat dough/bread quality or to import wheat from other countries to balance out wheat crop losses and qualities. However, these solutions offer rapid but not sustainable options and do not meet the growing consumers’ trend towards regional, nutritional, and clean-label foods. Another feasible approach to compensate for wheat crop losses and the altered wheat qualities may be offered by using flour blends of wheat and non-wheat grains for Western-style bakery products.

A considerable amount of literature has been published on the rheological and baking properties of wheat blends with alternative cereals. However, the existing body of this research has primarily focused on the nutritional enrichment of final products from such cereal blends, thus aiming to introduce the highest possible amounts of non-wheat (and, in most cases, gluten-free) grains into the flour blends. Until now, inadequate attention has been paid to the substitution of wheat in smaller amounts with the aim of optimizing or improving the rheological properties of doughs made from flour blends and their final product quality. While it is common knowledge that the lack of gluten affects baking properties negatively, less consideration has been given to the idea of whether low amounts of (gluten-free) cereals are advantageous in the baking of products from strong gluten wheat flours [6]. Promising evidence that small amounts of gluten-free cereal may have at least no negative effects on bread was pointed out by a study by Dube et al. [8], who concluded that a 10% sorghum addition to bread made from high-gluten wheat flour did not adversely influence bread properties.

Therefore, the general aim of this study was to compare how the addition of different non-wheat, gluten-free grains (sorghum, millet, amaranth, and buckwheat) affects the dough and baking quality of bakery products when blended with wheat flour. Emphasis was put on the use of the same methods and recipes for all flour blends, in order to allow a reliable comparison of eventual differences between the selected specialty grains, which so far has not been considered in previous publications. The decision criteria for selecting the non-wheat grains were their (existing or known) ability to be cultivated in Austria/middle Europe and their suitability to hot and dry climatic conditions. This was true for sorghum, millet, and amaranth; buckwheat, though, is minimally drought tolerant, but as it is more known in Austria than the beforementioned grains, it was included as well. All raw materials were thoroughly characterized for their chemical (ash, protein, starch, dietary fiber, and fat), physical (WAI and WSI), and pasting properties (RVA). For the experiments, wheat was blended with the respective grain to up to 30% for dough rheological determinations (Brabender Farinograph^®^ and Extensograph^®^, Brabender & Co. KG, Duisburg, Germany) and up to 40% in bakery products in 5% steps (2.5% steps for some specific conditions). The bakery products selected were a standardized yeast bread and a standardized sweet milk bread, the latter representing the fine bakery product range.

## 2. Results and Discussion

### 2.1. Chemical and Physical Characterization of the Flours

All raw materials were analyzed for the ash, protein, fat, and starch content, as well as for the water absorption index (WAI) and water-soluble index (WSI). These results are summarized in Table 1.

A noticeable lower ash content was found for wheat, which was expected as wheat was the only refined flour. Both red and white sorghum showed similar ash contents; the highest ash content was found in amaranth, followed by buckwheat. Decortication of the seeds only partially explains the difference in ash content, as amaranth was the only seed that was milled un-decorticated. Low protein contents were observed for red sorghum and millet. In white sorghum, it was higher, similar to buckwheat, and amaranth possessed the highest protein content. Among all alternative cereals, buckwheat showed the lowest fat content and was again highest in amaranth. The highest starch values were evaluated for wheat and buckwheat, while amaranth was characterized by the lowest amount, which was even lower than reported results from the literature [9]. All determined values lay in the range reported in the literature [9,10,11,12,13].

The water absorption index (WAI, defined as the amount of water absorbed by a defined amount of flour) is known for determining the hydration properties of a grain material. It is mostly influenced by the starch properties but also by fat and dietary fiber content. Both sorghum varieties had the highest WAI, which is most likely related to the fact that sorghum shows a huge bandwidth regarding starch granule sizes of 4–35 µm as reported by Zhu [14] in different sorghum varieties. In the present study, the average starch granule size was not measured. Furthermore, Beta et al. [15] mentioned that sorghum is known for being easily swollen in water and shows a fast water uptake. Regarding the specialty grains, amaranth had the lowest WAI, which may be explained by its low starch content, followed by millet and buckwheat. These results are in accordance with Culetu et al. [16]. High WSI values were found for both pseudocereals, in particular for amaranth, which may result from the fact that amaranth starch is characterized by low amylose contents and small granule sizes (0.8–2 µm, [17]). Regarding the cereals, WSI was higher for sorghum than for millet, which is in accordance with the results obtained by Di Cairano et al. [18].

### 2.2. Pasting Properties of the Flours

**Figure 1:**
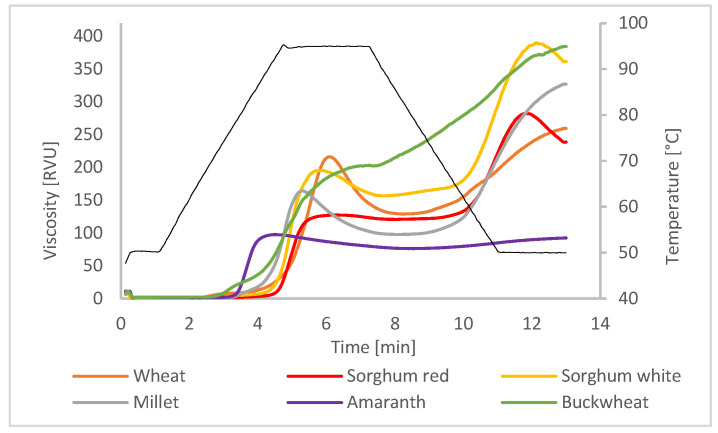
RVA pasting profile of selected gluten-free flours, compared with wheat flour (primary axis). Black line: RVA temperature profile (secondary axis).

The RVA pasting properties of the flours are presented in Figure 1.

Overall, pasting properties are mainly influenced by the starch content and its properties, such as amylose/amylopectin ratio, amylopectin chain lengths, and starch granule sizes.

The pasting temperatures (°C) in descending order for red sorghum, white sorghum, wheat, millet, amaranth, and buckwheat were 91.77 ± 0.5, 89.05 ± 0.5, 88.55 ± 0.5, 78.75 ± 0.5, 77.52 ± 0.1 and 73.17 ± 0.5, respectively. It is known that amylose is able to form complexes with lipids during thermal treatments, which can result in higher pasting temperatures [19]. This might be the reason for the low pasting temperature in amaranth, which is known to have low amylose content. However, in buckwheat, Quian [20] detected amylose content between 21.3–26.43%, so other causes may have been responsible for its low pasting temperature in this study. While both sorghum varieties had a higher pasting temperature than wheat, the pasting temperature of millet was lower. A higher pasting temperature for sorghum than for wheat was also reported by Awika [21] and Patil et al. [22].

Peak viscosity was highest in wheat (216.39 ± 12.11), followed by white sorghum (197.89 ± 13.88), millet (164.47 ± 0.82), red sorghum (127.58 ± 0.29), and amaranth (97.58 ± 6.22). For buckwheat, no defined peak viscosity was determinable. The grains showed significant differences with respect to breakdown, which was highest in wheat (87.33 ± 4.07), followed by millet (66.67 ± 3.18) and white sorghum (41.33 ± 9.06). Interestingly, the two sorghum varieties were significantly different from each other as only red sorghum showed almost no breakdown (6.83 ± 0.42), similar to the two pseudocereals. The breakdown of hot paste viscosity is an indicator of the stability of starch during heat treatment and is highly influenced by the inherent starch granules properties such as granule stability, crystallinity, or number of amorphous regions. A lower breakdown might also indicate that starch granule swelling and starch granule disruption occur at the same time [23]. Looking at the final viscosity, this parameter gives information on the retrogradation behavior of starch pastes upon cooling and is highly influenced by amylose content in particular. All grains, and especially buckwheat, showed a pronounced increase in final viscosity. According to Yoshimoto et al. [24], buckwheat possesses long amylopectin chains, which show great swelling properties. Therefore, buckwheat starch granules may have been more resistant to heat exposure, which resulted in a continuous viscosity increase upon cooling. In contrast, amaranth showed overall lower viscosity profile with a very low retrogradation tendency.

### 2.3. Rheological Properties

**Figure 2:**
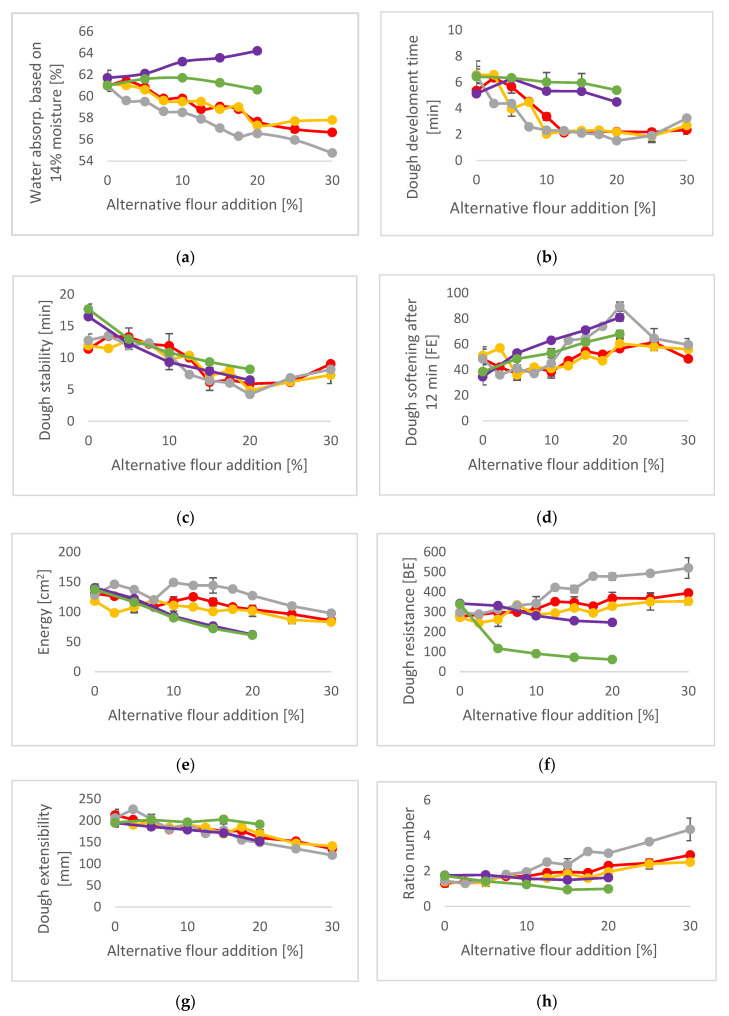
Farinograph^®^ and Brabender Extensograph^®^ data; Red: Red sorghum, Yellow: White sorghum, Grey: Millet, Purple: Amaranth, Green: Buckwheat; (**a**) Farinograph: Water absorption based on 14% moisture [%]; (**b**) Farinograph: Dough development time [min]; (**c**) Farinograph: Dough stability [min]; (**d**) Farinograph: Dough softening after 12 min [FE]; (**e**) Extensograph: Energy [cm^2^]; (**f**) Extensograph: Dough resistance [BE]; (**g**) Extensograph: Dough extensibility; (**h**) Extensograph: Ratio number.

In Figure 2, the rheological properties of wheat blended with either sorghum, millet, amaranth, or buckwheat are displayed.

In Figure 2a, it can be seen that with the increasing addition of sorghum and millet to wheat flour, the water absorption of the resulting doughs decreased continuously. An opposite behavior was observed for amaranth and slightly for buckwheat. Here the water absorption increased with higher amounts of amaranth/buckwheat flour, which might be explained by their difference in protein content but also their compositions. While sorghum and millet contain almost no water-soluble albumins, these represent the major protein class in amaranth, and buckwheat [25] analyses showed that the protein content was closely correlated with the water absorption (r = 0.8959). Additionally, it was observed that the ash content showed a positive correlation with water absorption (r = 0.9235). All correlation data can be taken from Supplementary Materials Table S1.

Dough development time (Figure 2b) decreased continuously with sorghum and millet addition, while it remained almost constant in the case of amaranth or buckwheat. Cereals possess a completely different protein quality than pseudocereals [26], which may have been the reason for the different behavior. With respect to dough stability and dough softening (Figure 2c,d), on the other hand, the differences between the cereals, millet, and sorghum, to the pseudocereals amaranth and buckwheat were less pronounced but revealed interesting results. While the addition of both pseudocereals continuously decreased dough stability and increased dough softening, the addition of millet and sorghum remained stable (until 12.5% addition) and at low additions of up to 5%, even showed an increased dough stability and a decreased dough softening. These rheological results give a first indication that the high gluten content in the used wheat flour was indeed too strong and resulted in a very compact dough where the gluten network was not able to develop properly. Addition of selected gluten-free cereals was able to reduce the extensive dough strength of the 100% wheat doughs. So far, previous studies did not report any increase in the dough stability after the addition of gluten-free cereals. However, in earlier times, wheat was not characterized by such a high or strong gluten content. At higher ratios, the addition of all gluten-free grains resulted in a weakening or dilution of the gluten network. It has to be considered that in this study, the specialty grains were added as wholegrain flours, and it is known that increased levels of dietary fiber interfere with the gluten proteins and are thus also responsible for a decrease in dough stability and increase in dough softening. This was also shown by Liu et al. [27], where the addition of wheat bran to wheat flour influenced both Farinograph parameters. No correlations were found between the dough development time, dough stability, dough softening, and chemical compositions (see Supplementary Materials Table S1).

Regarding the Extensograph data (Figure 2e–h), the differences between cereal and pseudocereal addition were even more visible. Energy and dough resistance decreased continuously with addition of amaranth and buckwheat, while in the case of sorghum and millet, the energy values remained almost stable, and dough resistance was even increased, in particular by millet addition. With respect to dough extensibility, the reference dough (100% wheat) showed a dough extensibility range between 195–212 mm, which is above the reference value of 150 mm for the selected wheat flour type [28]. Such high extensibility, resulting from a (too) high gluten content, can adversely affect the rheological properties and baking quality. The addition of all grains (except buckwheat) decreased this exceeding extensibility. With incorporation of 25% red/white sorghum or 20% millet or amaranth into wheat, a dough extensibility of 152 ± 1.4 mm, 147 ± 1.4 mm, 149 ± 4.2 and 152 ± 5.7, respectively, were achieved. In consequence, the ratio number of wheat flour showed a value between 1.3–1.8 and was decreased with the addition of amaranth and buckwheat and increased after the addition of sorghum and millet. An optimum ratio number for the used wheat flour is defined at 2.5 [28], which was reached with an addition of 30% sorghum or 15% millet. A decrease in the dough extensibility is favorably weakening the exceeding dough strength of the flour, which will be advantageous for baking, in particular for fine bakery products. Olckers et al. [29] found that flours with high protein contents, caused by heat and drought stress, resulted in a higher loaf volume after overmixing the dough, which reduced the dough strength and subsequently affected the bread quality positively.

### 2.4. Baking Properties

**Figure 3:**
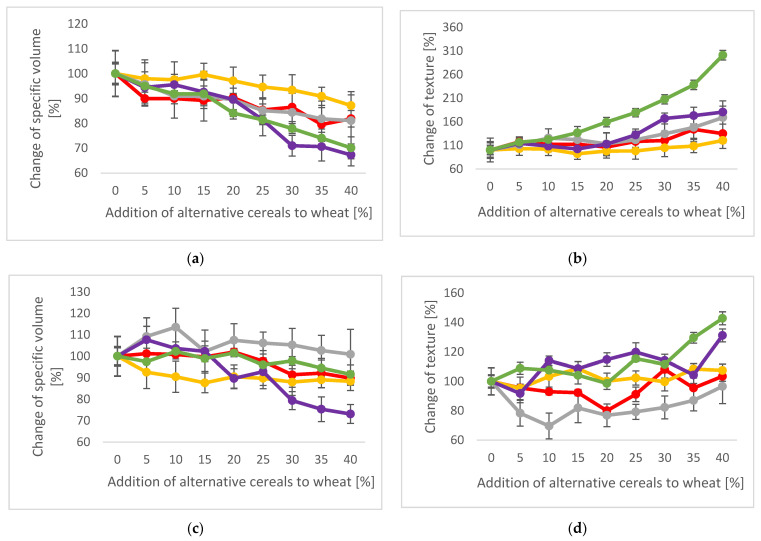
Physical baking properties; Red: Red sorghum, Yellow: White sorghum, Grey: Millet, Purple: Amaranth, Green: Buckwheat. The 100% wheat bread was defined as a reference. The data show the specific volume and crumb firmness compared with the reference (100% wheat). (**a**) Change of specific volume (%) of the standard wheat bread (SWB); (**b**) change of crumb firmness (%) of the SWB; (**c**) Change of the specific volume (%) of the sweet milk wheat bread (MWB); (**d**) Change of crumb firmness (%) of the MWB.

Selected results of the baking trials can be seen in Figure 3; detailed results, including statistical evaluation, are summarized in Supplementary Materials Tables S2 and S3.

In the standard wheat bread (SWB) baking trials, differences between the grains and also between varieties (within sorghum varieties) could be observed. Those results were already discussed in the previous publication of Rumler et al. [7]. While white sorghum caused no significant changes in bread volume and crumb firmness with additions of up to 20%, the addition of red sorghum, millet, and in particular, amaranth and buckwheat continuously reduced the volume already at low additions of 5 or 10%. Differences can be explained by the variation of chemical and physical properties, e.g., pasting properties, where white sorghum showed a high peak viscosity, unlike red sorghum (Figure 1). Xu et al. [30] reported that polyphenols can negatively affect wheat bread volumes. According to Speranza et al. [31], red sorghum varieties contained more polyphenols than white sorghum varieties, which may have contributed to the smaller volume of red sorghum breads. Overall, the pseudocereals influenced SBW volume the most, most likely caused by their different protein composition. Regarding texture, the addition of white sorghum improved the crumb firmness significantly; it was lowest with an addition of 15% and showed no differences within additions of up to 35%. However, this effect was not observed with the rest of the grains; here, crumb texture increased after a 5–10% addition. In general, sorghum had the lowest impact on crumb firmness, whereas buckwheat addition caused a three-fold firmer texture at 40% addition, which is in accordance with the retrogradation behavior during pasting (see Figure 1). In the case of amaranth, its low amylose content (comparable with the waxy type of cereals) might have been less detrimental to crumb texture, as suggested by the study of Yano et al. [32] using waxy rice flour. The baking loss was not markedly influenced by grain blending nor relative elasticity, which was only slightly decreased, except after buckwheat addition. Overall, the use of strong gluten wheat flour affected the quality of SBW to a lower extent compared with the sweet milk wheat bread (MWB). The addition of alternative grains negatively affected the SWB properties at amounts higher than 20%, opposite to what was seen in MWB.

As hypothesized for this study, the addition of alternative grains could positively influence the MWB final quality. Most successful in this respect was the blending with millet. At 5 and 10% millet addition, a significant increase in the MWB volume and a remarkable decrease in crumb firmness was observed, and no detrimental effects, even at 40% addition, were detected. Similar results were obtained by Maktouf et al. [33], who found a significant volume increase with an addition of 5% pearl millet, whereby in their study, an addition of 10% affected the bread volume again negatively. The authors assumed that the volume increase was connected to the high-fat content of millet but concluded that depending on the millet variety, formula, and percentage, millet additions can be useful to optimize wheat bread volume. After the addition of amaranth (5–15%), buckwheat (10%), and red sorghum (5–20%) MBW volume was increased, and only the white sorghum addition showed no beneficial effect. The texture was slightly improved at low levels of grain addition and worsened only at additions of more than 25% for most of them. Again, relative elasticity and baking loss were not affected after the addition of any alternative grain to a high extent (see Supplementary Materials Table S3).

### 2.5. Pore Analyses

**Figure 4:**
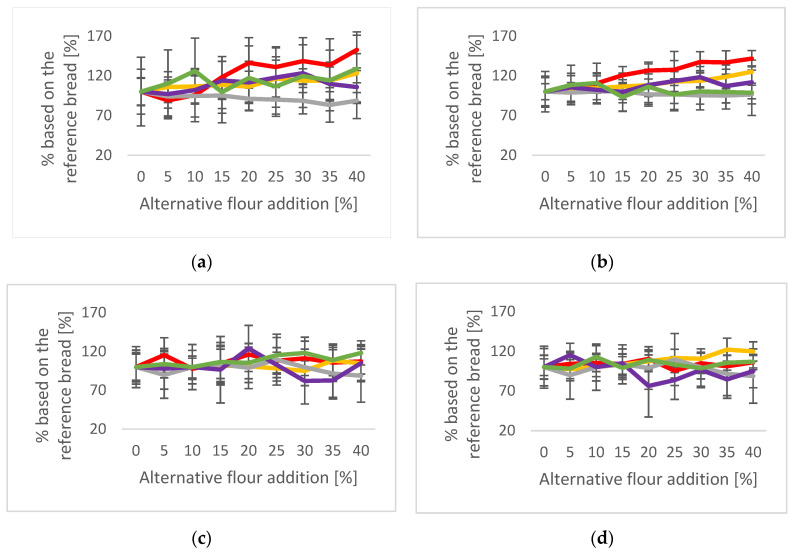
Pore pattern of the SWB and MWB; Red: Red sorghum, Yellow: White sorghum, Grey: Millet, Purple: Amaranth, Green: Buckwheat. This figure shows the pore properties of the different breads. The 100% wheat bread was defined as a reference. The data show the pore properties compared with the reference (100% wheat). (**a**) Change of the average pore size [%] of the SWB; (**b**) Change of the total pore area [%] of the SWB; (**c**) Change of the average pore size [%] of the MWB; (**d**) Change of the total pore area [%] of the MWB.

**Figure 5:**
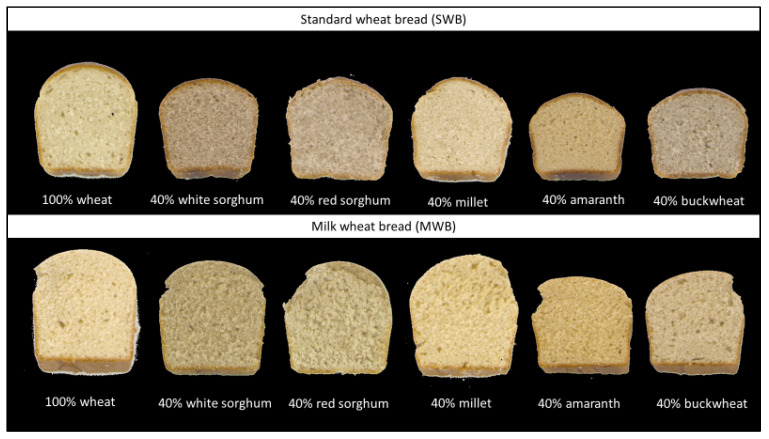
Bread slices of breads containing 40% alternative cereals and 60% wheat compared with the reference (100% wheat).

The crumb structure of a bread is an essential quality parameter. In Figure 4. the pore properties of the SWB and MWB are displayed, and pictures of selected bread slices are visible in Figure 5.

In SBW, sorghum addition and their variety significantly affected crumb pore sizes to a different extent (Figure 4a,c). Red sorghum addition decreased the pore size at low (5 and 10%) substitution levels, but significantly increased it at substitution levels of 15% and higher. These effects were obviously balanced by a higher number of pores at lower addition as the total pore area continuously increased. In other words, low addition of red sorghum resulted in more but smaller pores. At higher addition, the number of pores remained relatively constant, but their size increased. In contrast, the addition of white sorghum continuously increased pore size, at a fairly constant number, but was significantly higher only at 40%. Mwithiga and Sifunda [34] showed that the porosity was influenced by the sorghum variety. Millet addition caused a slight decrease in the average pore sizes at a higher number of pores. For amaranth and buckwheat, no clear trend could be defined.

In MBW, the porosity was generally less influenced by alternative grain addition compared with SBW. Sorghum addition slightly increased the pore area, mainly on account of the number of pores, and was more pronounced for white than red sorghum. In the case of millet addition, no clear changes were observed for both average pore size and total pore area (Figure 4b,d). An addition of 20% amaranth caused an increase in the average pore size and a decrease in the total pore area. Otherwise, no trend was visible with an increased addition. Buckwheat increased the average pore size significantly for the breads containing 40% buckwheat. However, the total pore area was already higher at addition of 10% buckwheat, indicating an increased number of pores.

### 2.6. Color Analyses

The color of the bread crumbs was measured and is shown in Supplementary Materials Table S4, but it can also be seen in Figure 5. As expected, the addition of alternative grains influenced most color values depending on the amount. Changes in color will be therefore described only for breads containing a level of 40% alternative grains (full data can be derived in the Supplementary Materials Table S4). The L*-value of the crumb decreased in both types of bread with any addition (except for white sorghum and millet in MBW). The crumb a*-value increased in the case of red sorghum (SWB) and amaranth (MWB) and decreased with the addition of buckwheat in SWB and by addition of red and sorghum, millet, and buckwheat in MWB. The crumb b*-values showed no significant changes after the addition of millet in SWB and of white sorghum and millet in MWB. All other breads appeared less yellowish.

## 3. Materials and Methods

### 3.1. Materials

Refined common wheat flour (*Triticum aestivum*) without food additives was obtained from GoodMills Österreich GmbH (Schwechat, Austria). The wet gluten content of the wheat flour was 33.3%, which characterized the flour as a high gluten flour. Wholegrain sorghum flours (*Sorghum bicolor* MOENCH, red variety *Armorik* and white variety *Ggolden*), wholegrain millet flour (*Pannicum miliaceum*, L.) and wholegrain buckwheat flour (*Fagopyrum esculentum* MOENCH) were purchased from Strobl Naturmühle (Linz-Ebelsberg, Austria). Amaranth (*Amaranthus* sp., variety *new green type*) was obtained from a farmer in lower Austria (harvested in 2020) and milled to wholegrain flour using a pin mill (Fa. Pallmann Maschinenfabrik—PXL 18, Zweibrücken, Germany). Sorghum varieties *Armorik* and *Ggolden*, millet, and buckwheat were harvested in the year 2019 and decorticated by abrasive milling prior to flour milling.

### 3.2. Physical and Chemical Characterization of the Raw Material

Ash, protein, starch, and fat contents were analyzed according to ICC or AACC standard methods as described by Rumler et al. [35]. The pasting properties of the raw flours were investigated by using a Rapid Visco Analyzer 4500 (PerkinElmer, Waltham, MA, USA) according to the ICC standard method 162. The water absorption index (WAI) and the water solubility index (WSI) were measured by following the method from Anderson et al. (1982) with slight modifications, as described by Rumler et al. [35]. Rheological flour properties were determined by using a Farinograph and Extensograph (Brabender^®^ GmbH & Co. KG, Duisburg, Germany) according to ICC standard methods 115/1 and 114/1, respectively. All analyses were carried out in triplicate, except for the Farinograph and Extensograph trials, which were performed in duplicate.

### 3.3. Baking Trials

- Standard wheat bread (SWB) preparation

For the standard wheat bread (SWB) preparation, a dough weight of 1000 g was produced based on the following recipe (% based on flour): 100% flour, 58% water, 3% dry yeast, 1.8% salt, and 1% sugar. All dry ingredients were homogenized at step 1 using a laboratory kneader (Bär Varimixer RN10 VL-2, Wodschow & Co., Brondby, Denmark). Afterward, the yeast (dissolved in 50 mL of the water) and the remaining water were added while kneading at step 2 for 30 s. Then, the dough was kneaded for 6 min and afterward molded by hand for 1 min. The obtained dough was put into a fermentation chamber (MANZ Backtechnik GmbH, Creglingen, Germany) set at 30 °C and 85% relative humidity (RH) for 30 min. After dough fermentation, the dough was separated into three 300 g portions, molded, folded, and rolled into a screw, which was placed into an oiled baking tin and submitted to a second fermentation period (30 °C, 85% RH) for 50 min. Finally, the breads were baked at 220 °C in a conventional deck oven (Model 60/rW, MANZ Backtechnik GmbH, Creglingen, Germany) for 25 min. After baking, breads were cooled at room temperature for 2 h and then stored at 20 °C, 50% RH in a climate chamber overnight until further analyses.

- Sweet milk wheat bread (MWB) preparation

For the sweet milk wheat bread (MWB) preparation, a dough weight of 1000 g was produced based on the following recipe (% based on flour): 100% flour, 50% milk, 6% yeast, 12% butter, 12% sugar, 6% fresh egg yolk and 1.5% salt. First, the milk was warmed up to 30 °C and homogenized with the yeast. Afterward, sugar, egg yolk, and salt were added, then homogenized with the flour by using a laboratory kneader (Bär Varimixer RN10 VL-2, Wodschow & Co., Brondby, Denmark) at step 2 for 2 min. Subsequently, the butter was added, and the dough was kneaded for a further 4 min, then molded by hand for 1 min. The obtained dough was placed into a fermentation chamber (MANZ Backtechnik GmbH, Creglingen, Germany) at 30 °C and 85% RH for 30 min, divided into 300g dough pieces, and fermented for a second period for 20 min. Finally, the doughs were baked at 170 °C in a conventional deck oven (Model 60/rW, MANZ Backtechnik GmbH, Creglingen, Germany) for 25 min. The cooled breads (2 h at room temperature) were stored at 20 °C and 50% RH relative humidity in a climate chamber overnight until further analyses. All SBW and MBW baking trials were carried out in triplicate, resulting in a total number of 9 bread loaves per formula.

### 3.4. Physical Properties of Bread

Specific volume: The volume (cm^3^) of the bread was evaluated by using the bread volume analyzer BVM 6600 (PerkinElmer, Waltham, MA, USA). Bread weight (g) was measured after the bread was fully cooled. Every loaf was measured in duplicate, resulting in a total number of 18 values per formula. The specific volume was calculated as displayed in Equation (1):

(1) $$\mathrm{Specific}\;\mathrm{volume}\;\left({\mathrm{cm}}^{3}/g\right)=\frac{\mathrm{Loaf}\;\mathrm{volume}\;\left({\mathrm{cm}}^{3}\right)}{\mathrm{Loaf}\;\mathrm{weight}\;\left(g\right)}$$

Volume yield: The volume yield (cm^3^/100 g flour) defines the loaf volume related to 100 g flour and is typically used by bakers. The calculation can be seen in Equation (2).

(2) $$\mathrm{Volume}\;\mathrm{yield}\;\left({\mathrm{cm}}^{3}/100\;g\;\mathrm{flour}\right)=\frac{\mathrm{Loaf}\;\mathrm{volume}\;\left({\mathrm{cm}}^{3}\right)}{\mathrm{Flour}\;\mathrm{amount}\;\mathrm{in}\;\mathrm{loaf}\;\left(g\right)}*100$$

Crumb firmness and relative elasticity: To characterize crumb firmness and relative elasticity, breads were analyzed by following the AACC Method 74–09.01 with some modifications. Three 3 cm thick slices per bread were cut and measured, resulting in 27 values per formula. A 50% compression test was carried out by using a Texture Analyzer (Model TA-XT plus C, Stable Micro systems™ Co., Godalming, UK), which was equipped with a 50 kg load cell and a cylindrical compression probe (SMS P/100). The settings were chosen according to Waziiroh et al. [36]: Test speed 0.5 mm/s, relaxation time 120 s, trigger force 10g, pre-test speed 1 mm/s, and post-test speed 10 mm/s. The crumb firmness was expressed as the maximum force (N) required to compress the crumb. The relative elasticity (%) was calculated by relating the residual force (N) at the end of the relaxation time to the maximum force (N) (Equation (3)).

(3) $$\mathrm{Relative}\;\mathrm{elasticity}\;\left(\%\right)=\frac{\mathrm{Residual}\;\mathrm{force}\;\mathrm{after}\;120\sec\;\left(N\right)}{\mathrm{Maximum}\;\mathrm{force}\;\left(N\right)}*100$$

Baking loss describes the amount of water lost during baking and was calculated as ratio of dough weight to loaf weight (Equation (4)).

(4) $$\mathrm{Baking}\;\mathrm{loss}\;\left(\%\right)=\frac{\mathrm{Dough}\;\mathrm{weight}\;\left(g\right)}{\mathrm{Loaf}\;\mathrm{weight}\;\left(g\right)}*100$$

Crumb and crust color analysis were carried out as described by Bender et al. [37]. Briefly, a DigiEye device (VeriVide, Leicester, UK) equipped with a D-90 Nikon camera (Tokyo, Japan) was used to characterize the color of the bread crumb and bread crust. For the expression of the results, CIE L*a*b* parameters were used.

For the determination of the crumb porosity, the procedure of Waziiroh et al. (2021) was applied using the Image J software. Values measured were the average pores size (mm) and the total pore area [%].

### 3.5. Statistical Analysis

All results were statistically analyzed by using Statgraphics Centurion 19.4.01 3 (Statpoint Technologies, Inc., Warrenton, VA, USA). Applying the ANOVA procedure and Fisher’s least significant test (α = 0.05). The correlation of data was carried out using the Pearson correlation test (α = 0.05).

## 4. Conclusions

Results of this study demonstrated that blending wheat flour with specialty gluten-free grains offers a new perspective for the use of (too) strong gluten wheat flour as affected by climate change. Rather than a decline in quality, in this study, it was seen that low levels of selected gluten-free cereals indeed could improve the rheology and (fine) baking properties of wheat flour, which was characterized by a high/strong gluten content, as determined by high dough extensibility and high dough strength. The addition of gluten-free grains (sorghum, millet, amaranth, and buckwheat) led to a relaxation of the dough; ideal dough extensibility values were observed when 25% white or red sorghum and 20% millet or amaranth were added to wheat. Baking trials demonstrated that the used gluten-free grains were able to improve final product quality (e.g., increased volume, decreased crumb firmness) when added at lower levels (5–15%, depending on the grain). While the used cereals, sorghum, and millet, could often be added at higher amounts of up to 40% without deteriorating the product quality significantly, the pseudocereals amaranth and buckwheat had a lower optimum level of addition (2.5–5%) without detrimental quality effects. This implies, in consequence, that if the aim is to replace wheat in higher amounts with alternative grains, e.g., to improve the nutritional properties of the final product, or to increase the biodiversity of grain production and consumption, the use of cereals such as millet or sorghum is recommendable. If the aim is to simply balance the overall flour blend quality, then gluten-free cereals and also pseudocereals (especially amaranth) can be incorporated, all at low levels of approx. 2.5–10%. Overall, wheat blending offers beneficial effects for the future in terms of increased biodiversity and improved nutrition and is thus a supportive tool to mitigate the effects of the ongoing climate change.

## Figures and Tables

**Table 1:** Ash, protein, fat, and starch contents of raw flours used for baking.

	Chemical Properties					Physical Properties	
Flour	Ash [%]	Protein [%]	Fat [%]	Starch [%]	Dietary Fiber [%]	WAI [g/g]	WSI [%]
Wheat	0.78 ± 0.03 ^a^	13.6 ± 1.15 ^c^	1.70 ± 0.14 ^a^	60.15 ± 0.80 ^d^	3.02 ± 0.31 ^b^	1.95 ± 0.02 ^a^	6.16 ± 0.04 ^d^
Red sorghum	1.56 ± 0.01 ^b^	6.83 ± 0.36 ^a^	4.28 ± 0.04 ^c^	49.74 ± 0.23 ^b^	8.49 ± 1.09 ^d^	2.56 ± 0.01 ^f^	5.53 ± 0.01 ^c^
White sorghum	1.55 ± 0.02 ^b^	10.15 ± 0.02 ^b^	4.07 ± 0.38 ^b,c^	55.15 ± 2.51 ^c^	10.07 ± 0.94 ^e^	2.51 ± 0.01 ^e^	4.84 ± 0.05 ^b^
Millet	1.83 ± 0.04 ^c^	8.01 ± 0.01 ^a,b^	6.42 ± 0.05 ^d^	57.34 ± 1.96 ^c^	1.01 ± 0.31 ^a^	2.28 ± 0.02 ^c^	1.95 ± 0.05 ^a^
Amaranth	2.85 ± 0.04 ^e^	17.23 ± 0.65 ^d^	8.63 ± 1.44 ^e^	45.40 ± 1.09 ^a^	5.47 ± 0.23 ^c^	2.24 ± 0.02 ^b^	12.17 ± 0.33 ^f^
Buckwheat	2.18 ± 0.11 ^d^	10.47 ± 3.71 ^b^	2.99 ± 0.09 ^b^	60.80 ± 1.65 ^d^	3.59 ± 0.46 ^b^	2.42 ± 0.02 ^d^	7.82 ± 0.47 ^e^

All results are expressed in dry matter. Means (n = 3 ± standard deviation) with different superscript letters are significantly different at *p* < 0.05 according to Fisher’s Least Significant Difference test.

## Data Availability

All data are included within the article or within the Supplementary Material.

## Supplementary Materials

The following supporting information can be downloaded at: https://www.mdpi.com/article/10.3390/plants12030492/s1, Table S1: Pearson correlation of chemical and rheological data; Table S2: Physical baking properties (Specific volume, volume yield, texture, baking loss) of the SWB including alternative cereal (red sorghum, white sorghum, millet, amaranth, buckwheat); Table S3: Physical baking properties (Specific volume, volume yield, texture, baking loss) of the MWB including alternative cereal (red sorghum, white sorghum, millet, amaranth, buckwheat); Table S4: SWB and MWB crumb colors (L*a*b* values).
