# Supplementary material for: Mitigating the Effect of Climate Change within the Cereal Sector: Improving Rheological and Baking Properties of Strong Gluten Wheat Doughs by Blending with Specialty Grains
Source: Plants (Basel). 2023 Jan 21;12(3):492. doi: 10.3390/plants12030492 (PMC9919387; doi:10.3390/plants12030492)
Supplement: Supplementary file 1 [file plants-12-00492-s001.zip › plants-2067209-supplementary.pdf]

Table S1. Pearson correlation of chemical and rheological data.

		Water absoption [%]	Dough developmemt time [min]	Dough stability [min]	Dough softening [BU]	Energy [cm2]	Dough resistance [BU]	Extensibility [mm]	Ratio number
**Ash**	*r*	0.9235	0.5038	0.7301	0.5277	-0.7474	-0.5656	-0.1249	-0.5051
	p	0.0251	0.3868	0.1614	0.3607	0.1465	0.3204	0.8414	0.3854
**Protein**	*r*	0.8959	0.3408	0.6217	0.3489	-0.7242	-0.5831	-0.1836	-0.5167
	p	0.0397	0.5747	0.2628	0.5649	0.1665	0.3021	0.7675	0.3727
**Fat**	*r*	0.4844	-0.2751	-0.0271	0.7096	-0.0544	0.1832	-0.8098	0.2654
	p	0.4083	0.6542	0.9655	0.1794	0.9308	0.768	0.0967	0.6661
**Starch**	*r*	-0.5213	0.0932	0.0277	0.0154	0.1765	0.0123	0.7321	-0.0723
	p	0.3677	0.8815	0.9647	0.9804	0.7764	0.9844	0.1596	0.908
**Dietary fiber**	*r*	-0.0421	-0.0801	-0.167	-0.8331	-0.0395	-0.1576	-0.0065	-0.1505
	p	0.9464	0.8981	0.7883	0.0798	0.9498	0.8002	0.9917	0.8091

Table S2. Physical baking properties (Specific volume, volume yield, texture, baking loss) of the SWB including alternative cereal (red sorghum, white sorghum, millet, amaranth, buckwheat).

	Addition [%]	0	5	10	15	20	25	30	35	40
Red sorghum	Specific volume [ml/g]	2.59±0.24f	2.33±0.06de	2.41±0.05e	2.31±0.05cd	2.34±0.04de	2.21±0.11b	2.24±0.13bc	2.06±0.14a	2.12±0.07a
	Parameter compared to control [%]	100±9	90±3	90±2	89±2	90±2	85±5	86±6	80±7	82±3
White sorghum	Specific volume [ml/g]	2.41±0.11e	2.36±0.18de	2.35±0.17de	2.4±0.11e	2.34±0.13de	2.28±0.11cd	2.25±0.14bc	2.19±0.08b	2.1±0.09a
	Parameter compared to control [%]	100±5	98±8	98±7	100±5	97±6	95±5	93±6	91±4	87±4
Millet	Specific volume [ml/g]	2.75±0.25e	2.63±0.23de	2.5±0.22cd	2.5±0.25cd	2.46±0.19bc	2.34±0.12ab	2.32±0.18a	2.25±0.16a	2.23±0.26a
	Parameter compared to control [%]	100±9	95.64±9	90.91±9	90.91±10	89.45±8	85.09±5	84.36±8	81.82±7	81.09±12
Amaranth	Specific volume [ml/g]	2.69±0.11e	2.54±0.16d	2.57±0.08d	2.49±0.12cd	2.41±0.11c	2.19±0.14b	1.91±0.08a	1.9±0.11a	1.81±0.08a
	Parameter compared to control [%]	100±4	94.42±6	95.54±3	92.57±5	89.59±5	81.41±6	71±4	70.63±6	67.29±4
Buckwheat	Specific volume [ml/g]	2.58±0.1h	2.45±0.1g	2.37±0.03c	2.37±0.14c	2.17±0.03e	2.1±0.04d	2.01±0.04c	1.91±0.07b	1.81±0.08a
	Parameter compared to control [%]	100±4	94.96±4	91.86±1	91.86±6	84.11±1	81.4±2	77.91±2	74.03±4	70.16±4
Red sorghum	Volume yield [ml/100g flour]	380.4±27.14f	347.36±9.12de	358.32±6.29e	344.96±5.79cd	347.6±6.27d	328.45±18.19b	335.55±18.77bc	308.56±21.02a	317.12±11.46a
	Parameter compared to control [%]	100±7	91±3	94±2	91±2	91±2	86±6	88±6	81±7	83±4
White sorghum	Volume yield [ml/100g flour]	361.08±16.16f	353.19±24.83ef	351.27±22.84def	356.5±18.16ef	347.81±17.7de	339.41±14.89cd	333.24±19.15bc	325.8±12.89b	313.33±14.23a
	Parameter compared to control [%]	100±4	98±7	97±7	99±5	96±5	94±4	92±6	90±4	87±5
Millet	Volume yield [ml/100g flour]	392.85±33.53d	375.95±30.97de	360.45±30.36bc	359.69±32.99bc	354.42±27.49b	336.28±15.51a	331.39±22.68a	323.6±18.68a	319.53±29.45a
	Parameter compared to control [%]	100±9	95.7±8	91.76±8	91.56±9	90.22±8	85.6±5	84.36±7	82.37±6	81.2±9
Amaranth	Volume yield [ml/100g flour]	392.85±33.53f	375.95±30.97ab	360.45±30.36e	359.69±32.99e	354.42±27.49d	336.28±15.51c	331.39±22.68b	323.6±18.68ab	319.53±29.45a
	Parameter compared to control [%]	100±3	95±4	95±3	92±4	88±5	81±7	71±4	71±5	67±4
Buckwheat	Volume yield [ml/100g flour]	380.41±14.42h	359.5±13.96g	350.86±5.51f	347.58±18.5f	319.62±4.16e	310.22±4.53d	296.71±4.61c	281.24±10.17b	263.81±8.7a
	Parameter compared to control [%]	100±4	95±4	92±2	91±5	84±1	82±1	78±2	74±4	69±3
Red sorghum	Crumb texture [N]	28.58±7.22a	33.48±3.62bc	32±2.49bc	32.07±3.6bc	30.36±3.91ab	33.76±7.4bc	34.33±6.92c	41.1±10.08d	38.51±7.71d
	Parameter compared to control [%]	100±25	117±11	112±8	112±11	106±13	118±22	120±20	144±26	135±20
White sorghum	Crumb texture [N]	36±3.13abc	37±5.25bc	36±4.86bc	33±3.93a	35±5.16ab	35±6.11ab	37±7.06bc	39±5.32c	43±7.32d
	Parameter compared to control [%]	100±9	103±14	102±13	92±12	98±15	98±18	105±19	108±14	120±17
Millet	Crumb texture [N]	26.77±4.77a	29.8±3.95ab	33.55±6.61bc	32.61±6.37bc	30.24±6.95ab	32.56±5.25bc	35.94±9.7cd	39.56±9.12d	45.17±16.04e
	Parameter compared to control [%]	100±18	111.32±13	125.33±20	121.82±20	112.96±23	121.63±16	134.25±27	147.78±23	168.73±36
Amaranth	Crumb texture [N]	22.96±3.46a	26.23±2.61c	24.85±2.93abc	23.48±3.1ab	25.78±6.27bc	30.29±3.82d	38.29±4.37e	39.68±7.03ef	41.41±5.21f
	Parameter compared to control [%]	100±15	114±10	108±12	102±13	112±24	132±13	167±11	173±18	180±13
Buckwheat	Crumb texture [N]	30.66±3.11a	35.68±3.8b	37.42±2.9b	41.86±5.63c	48.77±4.92d	54.91±4.76e	63.57±6.23f	72.95±7.22g	92.21±9.36h
	Parameter compared to control [%]	100±10	116±11	122±8	137±13	159±10	179±9	207±10	238±10	301±10
Red sorghum	Relative Elasticity [%]	54.75±1.69g	53.49±1.26f	52.85±1.25e	52.07±1.33d	51.79±0.84d	50.11±1.29bc	50.25±0.9c	49.6±0.91ab	49.35±0.89a
	Parameter compared to control [%]	100±3	98±2	97±2	95±3	95±2	92±3	92±2	91±2	90±2
White sorghum	Relative Elasticity [%]	54.18±1.12a	52.98±1.2b	51.97±1.27c	51.97±1.27c	51.15±0.72d	50.51±0.99f	50.5±0.79f	49.97±0.82fg	49.56±0.9g
	Parameter compared to control [%]	100±2	98±2	96±2	96±2	94±1	93±2	93±2	92±2	91±2
Millet	Relative Elasticity [%]	54.8±1.95f	53.16±1.57e	52.18±1.06d	51.78±1.43cd	51.8±1.05cd	51.57±1.06cd	51.02±1.36bc	50.62±1.52b	49.31±2.49a
	Parameter compared to control [%]	100±4	97±3	95±2	94±3	95±2	94±2	93±3	92±3	90±5
Amaranth	Relative Elasticity [%]	54.72±2.12f	52.57±1.55e	51.4±1.69d	50.45±1.52d	51.31±2.84d	48.88±1.91c	47.68±1.93b	47.04±1.63ab	46.39±1.45a
	Parameter compared to control [%]	100±4	96±3	94±3	92±3	94±6	89±4	87±4	86±3	85±3
Buckwheat	Relative Elasticity [%]	53.93±0.79f	53.12±0.9ef	52.11±1.11cd	51.87±0.99bcd	52.14±0.9d	51.46±1.03ab	51.45±1.22ab	51.31±1.15a	51.59±0.85abc
	Parameter compared to control [%]	100±15	114±10	108±12	102±13	112±24	132±13	167±11	173±18	180±13
Red sorghum	Baking loss [%]	11.07±2.13b	9.31±0.29a	9.08±0.93b	8.97±0.73a	9.29±0.69a	9.42±0.64a	8.77±0.11a	8.74±0.31a	9.06±0.8a
	Parameter compared to control [%]	100±19	84±3	82±10	81±8	84±7	85±7	79±1	79±4	82±9
White sorghum	Baking loss [%]	8.91±0.18a	8.83±0.51a	9.09±0.89abc	9.63±0.47d	9.52±0.31cd	9.36±0.23bcd	9.62±0.45d	9.44±0.5bcd	9.03±0.44ab
	Parameter compared to control [%]	100±2	99±6	102±10	108±5	107±3	105±2	108±5	106±5	101±5
Millet	Baking loss [%]	11.76±3.66a	11.78±3.67a	11.03±3.45a	11.14±3.47a	11.33±3.54a	11.46±3.57a	11.67±3.64a	11.26±3.51a	11.63±3.73a
	Parameter compared to control [%]	100±31	100±31	94±31	95±31	96±31	97±31	99±31	96±31	99±32
Amaranth	Baking loss [%]	11.81±2.9ab	11.5±2.86b	12.96±0.98a	12.76±0.64ab	13.09±0.51a	12.66±0.5ab	11.77±0.69ab	12.17±0.74ab	11.82±0.75ab
	Parameter compared to control [%]	100±25	97±25	110±8	108±5	111±4	107±4	100±6	103±6	100±6
Buckwheat	Baking loss [%]	10±0.6abc	10.3±0.36c	9.67±0.49a	10.2±0.65bc	9.91±0.3abc	9.8±0.62ab	9.95±0.62abc	10.04±0.29abc	9.77±0.5ab
	Parameter compared to control [%]	100±6	103±3	97±5	102±6	99±3	98±6	100±6	100±3	98±5

Different superscript letters denote significant differences within the same row (evaluated by the Fisher's Least Significant Difference test)

Table S3. Physical baking properties (Specific volume, volume yield, texture, baking loss) of the MWB including alternative cereal (red sorghum, white sorghum, millet, amaranth, buckwheat).

	Addition [%]	0	5	10	15	20	25	30	35	40
Red sorghum	Specific volume [ml/g]	2.55±0.14bc	2.58±0.17bc	2.57±0.13bc	2.54±0.16bc	2.6±0.15bc	2.49±0.07b	2.33±0.13a	2.35±0.13a	2.29±0.13a
	Parameter compared to control [%]	100±5	101±7	101±5	100±6	102±6	98±3	91±6	92±6	90±6
White sorghum	Specific volume [ml/g]	2.83±0.08d	2.62±0.12c	2.56±0.16bc	2.48±0.14a	2.56±0.1bc	2.54±0.1ab	2.49±0.12ab	2.52±0.05ab	2.5±0.04ab
	Parameter compared to control [%]	100±3	93±5	90±6	88±6	90±4	90±4	88±5	89±2	88±2
Millet	Specific volume [ml/g]	2.29±0.2a	2.5±0.11c	2.6±0.15d	2.34±0.06ab	2.46±0.07c	2.43±0.27bc	2.41±0.08bc	2.35±0.13ab	2.31±0.19a
	Parameter compared to control [%]	100±9	109.17±4	113.54±6	102.18±3	107.42±3	106.11±11	105.24±3	102.62±6	100.87±8
Amaranth	Specific volume [ml/g]	2.23±0.15cde	2.4±0.05e	2.31±0.1de	2.28±0.08cde	2±0.37bc	2.07±0.26bcd	1.77±0.38ab	1.68±0.29a	1.63±0.34a
	Parameter compared to control [%]	100±7	107.62±2	103.59±4	102.24±4	89.69±19	92.83±13	79.37±21	75.34±17	73.09±21
Buckwheat	Specific volume [ml/g]	2.73±0.12def	2.66±0.13cd	2.79±0.22f	2.7±0.1de	2.77±0.1ef	2.62±0.1bc	2.67±0.11cd	2.58±0.07b	2.5±0.04a
	Parameter compared to control [%]	100±4	97.44±5	102.2±8	98.9±4	101.47±4	95.97±4	97.8±4	94.51±3	91.58±2
Red sorghum	Volume yield [ml/100g flour]	454.99±22.88bc	461.89±29.23bc	459.51±22.02bc	455.13±26.54bc	466.37±26.55c	446.16±13.31b	415.52±22.47a	419.99±24.69a	409.65±23.47a
	Parameter compared to control [%]	100±5	102±6	101±5	100±6	103±6	98±3	91±5	92±6	90±6
White sorghum	Volume yield [ml/100g flour]	505.14±14.94c	470.31±22.99ab	456.85±30.95a	458.38±14.03ab	472.86±30.6b	467.26±25.31ab	461.52±29.33ab	466.54±18.6ab	461.74±20.19ab
	Parameter compared to control [%]	100±3	93±5	90±7	91±3	94±6	93±5	91±6	92±4	91±4
Millet	Volume yield [ml/100g flour]	411.11±28.07ab	442.26±23.43d	461.11±27.45e	405.84±11.25a	431.98±8.12cd	424.41±46.31bc	424.34±15.74bc	412.33±24.06ab	405.18±35.12a
	Parameter compared to control [%]	100±7	107.58±5	112.16±6	98.72±3	105.08±2	103.24±11	103.22±4	100.3±6	98.56±9
Amaranth	Volume yield [ml/100g flour]	411.11±28.07bc	442.26±23.43d	461.11±27.45cd	405.84±11.25cd	431.98±8.12bc	424.41±46.31cd	424.34±15.74ab	412.33±24.06a	405.18±35.12a
	Parameter compared to control [%]	100±7	108±1	102±4	102±4	89±18	93±13	79±21	75±17	73±21
Buckwheat	Volume yield [ml/100g flour]	467.06±20.5def	455.74±24.12cd	477.02±37.51f	462.76±15.51de	471.55±14.91ef	448.97±15.1bc	458.34±14.28cd	442.64±8.07ab	431.53±6.97a
	Parameter compared to control [%]	100±4	98±5	102±8	99±3	101±3	96±3	98±3	95±2	92±2
Red sorghum	Crumb texture [N]	20.48±2.51cd	19.56±1.66bc	19.03±2.36bc	18.91±2.64b	16.39±3.23a	18.67±2.62b	22.14±3.77e	19.55±2.67bc	21.18±3.63de
	Parameter compared to control [%]	100±12	96±8	93±12	92±14	80±20	91±14	108±17	95±14	103±17
White sorghum	Crumb texture [N]	17±1.74ab	16±2.34a	18±3.53bc	18±2.87c	17±1.88ab	17±1.72bc	17±1.64ab	18±1.31c	18±1.5c
	Parameter compared to control [%]	100±10	95±14	103±20	109±16	100±11	102±10	100±10	108±7	107±8
Millet	Crumb texture [N]	25.45±5.76d	19.94±4.28ab	17.73±3.87a	20.85±4.55b	19.58±2.62ab	20.17±3.37ab	20.95±3.77b	22.15±6.53bc	24.58±9.35cd
	Parameter compared to control [%]	100±23	78.35±21	69.67±22	81.93±22	76.94±13	79.25±17	82.32±18	87.03±29	96.58±38
Amaranth	Crumb texture [N]	20.96±2.31ab	19.24±1.78a	23.89±3.88cd	22.77±4.41bcd	24.05±7.05cd	25.11±4.13de	23.93±3.71de	21.87±2.88abc	27.49±5.63e
	Parameter compared to control [%]	100±11	92±9	114±16	109±19	115±29	120±16	114±16	104±13	131±20
Buckwheat	Crumb texture [N]	17.14±3.55a	18.66±3.49abc	18.44±4.59abc	17.85±2.6ab	16.91±1.87a	19.77±2.42c	19.11±2.61bc	22.2±2.66d	24.47±5.03e
	Parameter compared to control [%]	100±21	109±19	108±25	104±15	99±11	115±12	111±14	130±12	143±21
Red sorghum	Relative Elasticity [%]	46.65±1.14d	46.16±1.21d	45.57±0.97c	45.01±1.3bc	44.88±1.08b	44.54±1.04ab	44.88±0.83b	44.31±0.89a	44.19±0.84a
	Parameter compared to control [%]	100±2	99±3	98±2	96±3	96±2	95±2	96±2	95±2	95±2
White sorghum	Relative Elasticity [%]	47.01±1.06f	45.31±1.07bcd	45.78±0.93de	45.6±0.84cde	45.84±0.82e	45.41±0.82bcde	45.01±0.86ab	45.17±0.71ab	44.62±0.83a
	Parameter compared to control [%]	100±2	96±2	97±2	97±2	98±2	97±2	96±2	96±2	95±2
Millet	Relative Elasticity [%]	42.37±3.98ab	43.06±1.15b	42.93±0.93b	42.62±1.1b	42.88±1.05b	43.02±0.96b	42.31±1ab	42.75±1.2ab	41.94±1.46a
	Parameter compared to control [%]	100±9	102±3	101±2	101±3	101±2	102±2	100±2	101±3	99±3
Amaranth	Relative Elasticity [%]	44.86±1.19c	44.41±0.97bc	44.15±1.06bc	44.27±0.89bc	41.07±2.61a	40.35±10.36a	42.25±1.49ab	42.03±1.67ab	41.48±2a
	Parameter compared to control [%]	100±3	99±2	98±2	99±2	92±6	90±26	94±4	94±4	92±5
Buckwheat	Relative Elasticity [%]	45.74±0.89cd	45.66±0.68cd	45.85±0.66d	45.95±0.72d	45.9±0.61d	45.42±0.77b	44.81±0.73b	44.46±0.76b	44.05±1.01a
	Parameter compared to control [%]	100±11	92±9	114±16	109±19	115±29	120±16	114±16	104±13	131±20
Red sorghum	Baking loss [%]	4.92±0.56b	4.61±0.5ab	4.62±0.26ab	4.52±0.33b	4.45±0.18a	4.67±0.3ab	4.76±0.41ab	4.62±0.3ab	4.67±0.27ab
	Parameter compared to control [%]	100±11	94±11	94±6	92±7	90±4	95±6	97±9	94±6	95±6
White sorghum	Baking loss [%]	4.85±0.39abc	4.54±0.26a	4.88±0.4bc	4.66±0.54ab	5.27±0.29d	5.1±0.22cd	4.89±0.38bc	5.04±0.37de	5.03±0.14cd
	Parameter compared to control [%]	100±8	94±6	101±8	96±12	109±6	105±4	101±8	104±7	104±3
Millet	Baking loss [%]	5.63±0.71a	5.57±1.05a	5.59±1.36a	7.58±0.51c	6.51±1.26ab	6.83±1.5cd	6.29±1.12ab	6.47±1.08ab	6.33±0.83ab
	Parameter compared to control [%]	100±13	99±19	99±24	135±7	116±19	121±22	112±18	115±17	112±13
Amaranth	Baking loss [%]	7.87±1.19ab	7.7±1.52a	9.14±0.25c	8.73±0.53bc	7.93±0.95ab	8.01±0.49ab	8.67±0.33bc	8.25±0.12abc	8.4±0.54abc
	Parameter compared to control [%]	100±15	98±20	116±3	111±6	101±12	102±6	110±4	105±1	107±6
Buckwheat	Baking loss [%]	8.74±0.75b	8.61±0.71b	8.73±0.32b	8.52±0.62ab	9.04±0.53b	8.56±0.6b	8.37±1.02ab	8.43±1.08ab	7.75±1.27a
	Parameter compared to control [%]	100±9	99±8	100±4	97±7	103±6	98±7	96±12	96±13	89±16

Different superscript letters denote significant differences within the same row (evaluated by the Fisher's Least Significant Difference test

Table S4. SWB and MWB crumb colors (L*a*b* values).

Parameter	Cereal	0%	5%	10%	15%	20%	25%	30	35	40
SWB - L* Crumb	Sorghum Armorik	70.97±8.38a	66.8±9.89bc	64.71±10.87bc	67.72±2.22ab	66.63±1.47bc	60.02±5.93ab	63.42±3.11cd	58.47±5.35e	57.46±5.83e
	Sorghum Ggolden	75.39±2.04a	73.78±1.63b	73.46±1.28b	71.72±1.42c	70.55±1.33ef	71.06±1.91cd	69.55±1.82fg	68.61±2.98g	68.6±3.06g
	Millet	75.08±2.3ab	75.17±2.38ab	76.01±2.5bc	74.78±3.21a	76.75±2.28c	75.08±1.73ab	76.08±1.47bc	76.58±1.63c	76.09±2.04c
	Amaranth	71.31±1.5a	68.12±1.36b	65.24±3.07c	64.24±0.92cd	63.82±0.93d	63.63±0.29d	60.07±1.38ef	61.11±0.67e	59.72±1.22f
	Buckwheat	76.61±4.03a	74.32±2.28b	72.18±2.9c	69.19±3.19d	69.18±2.07d	68.21±3.12d	65.75±3.08e	63.63±1.53f	62.49±1.82f
SWB - a* Crumb	Sorghum Armorik	6.71±6.1a	7.04±4.88ab	7.56±4.58ab	6.61±0.56a	7.14±0.68ab	8.78±1.6cd	8.49±0.9bcd	9.14±1.14cd	9.54±0.96d
	Sorghum Ggolden	3.88±0.39a	4.19±0.5ab	4.49±0.57bc	4.84±0.56cd	5.15±0.65d	5.57±0.73e	6.03±0.79f	6.4±1.01fg	6.64±0.99g
	Millet	3.99±0.92a	3.95±0.85ab	3.76±0.83abc	3.95±0.8ab	3.54±0.66c	3.51±0.73c	3.57±0.49bc	3.64±0.58abc	3.61±0.48abc
	Amaranth	5.01±0.85a	5.95±0.48b	6.16±0.34b	6.94±0.39c	7.66±0.55d	8.22±0.34e	8.85±0.75f	8.76±0.27f	9.12±0.43f
	Buckwheat	4.39±0.48ab	4.18±0.43ab	4.45±0.49b	4.78±0.57c	4.6±0.53bc	4.81±0.5c	5.1±0.47d	5.58±0.41e	5.56±0.4e
SWB - b* Crumb	Sorghum Armorik	23.17±4.83a	21.88±3.11bc	21.22±2bc	20.26±0.54cd	19.63±0.73d	19.92±0.96d	20.12±1.08ab	19.81±0.91d	20.2±0.75cd
	Sorghum Ggolden	21.63±0.81a	20.99±0.9b	20.9±0.81b	20.1±0.8c	19.78±0.65cd	19.82±0.82cd	19.55±0.75d	19.62±0.64d	19.48±0.62d
	Millet	20.37±1.09a	21.14±1.25b	21.26±1.16bc	21.38±0.99bc	21.66±1bcd	21.6±1.21bcd	21.89±0.93cd	22.19±1.25de	22.65±1.71e
	Amaranth	24.31±0.44a	25.33±0.54b	25.55±0.6b	25.97±0.36b	27.13±1.05c	27.45±1.58cd	28.14±1.05cd	27.72±1.33cd	27.66±1d
	Buckwheat	21.39±0.74a	20.19±0.86b	19.73±0.75c	18.92±1.02d	18.36±0.6e	18.1±0.79ef	17.72±1.13f	17±0.66g	16.53±0.45h
MWB - L* Crumb	Sorghum Armorik	80.34±1.3a	77.18±0.95b	74.37±1.46c	73.9±1.61c	71.5±1.56d	69.49±1.61e	67.71±1.1f	66.13±1.45g	65.5±1.53g
	Sorghum Ggolden	79.95±1.17a	78.29±1.48b	77.32±1.18c	75.37±1.72d	75.08±0.81d	73.7±1.28e	73.09±1.01e	71.4±1.55f	68.96±1.57g
	Millet	83.17±5.08bc	83.42±5.87bc	83.52±4.78c	81.34±1.79ab	82.8±3.9bc	84.26±3.96c	84.29±4.35c	80.18±2.86a	80.21±2.06a
	Amaranth	82.48±2.3f	81.23±2.18f	78.68±2.37e	75.37±2.29d	73.8±2.09bc	74.76±1.88cd	72.96±1.61b	72.47±0.98b	70.15±3.21a
	Buckwheat	80.5±2.35a	80.12±1.54a	79.13±2.44b	76.72±1.3c	75.01±1.57d	74.39±1.52de	73.71±1.01ef	72.92±1.04f	70.11±1.19g
MWB - a* Crumb	Sorghum Armorik	4.43±0.43a	4.97±0.57b	5.67±0.54c	6.16±0.64d	7±0.43e	7.59±0.6f	7.86±0.51fg	8.1±0.69g	8.56±0.59h
	Sorghum Ggolden	3.95±0.42a	4.35±0.56b	4.91±0.51c	5.23±0.5d	5.63±0.79e	5.82±0.64ef	6.07±0.61fg	6.24±0.63gh	6.43±0.52h
	Millet	3.79±0.88abc	3.8±0.89abc	4.03±0.72bcd	4.37±0.5d	3.67±0.85ab	3.76±0.78abc	3.46±0.98a	4.11±0.42cd	4.34±0.43d
	Amaranth	4.25±0.54a	4.54±0.42b	4.76±0.32bc	5.03±0.5c	5.55±0.37d	5.63±0.46d	5.97±0.46e	6.5±0.48f	6.87±0.4g
	Buckwheat	4.38±0.57a	4.34±0.5a	4.66±0.53b	4.67±0.49b	4.86±0.47bc	5.06±0.53c	5.42±0.47d	5.56±0.43de	5.71±0.54e
MWB - b* Crumb	Sorghum Armorik	26.42±0.7a	25.36±1.02b	24.95±0.7c	24.08±0.95d	23.69±0.69d	22.81±0.71e	22.59±0.46e	21.86±0.63f	22.01±0.62f
	Sorghum Ggolden	26.02±0.81a	25.76±0.36ab	25.45±0.58b	25±0.58c	24.64±0.71c	24.22±0.58d	23.44±0.42e	23.98±1.28d	22.79±0.68f
	Millet	25.7±1.13a	25.72±0.78a	27.05±0.84b	27.61±1.02b	27.28±1.51b	27.37±1.02b	27.63±1.38b	28.58±0.87c	29.48±1.33d
	Amaranth	25.27±0.72a	26.12±0.5b	26.01±0.45b	26.68±0.58c	27.17±0.63cd	26.85±0.95cd	26.45±0.71bc	27.46±0.59de	27.69±1.12e
	Buckwheat	25.55±0.74ab	25.68±0.78a	25.13±0.82bc	24.77±0.47cd	24.54±0.55d	24.35±0.42d	23.59±0.94e	23.41±0.99e	22.8±1.07f
